# Medical interns and health challenges: insights into physical inactivity, sleep disruption, and body metrics

**DOI:** 10.3389/frhs.2026.1735424

**Published:** 2026-03-18

**Authors:** Ricardo Salas-Flores, Raúl De León-Escobedo, Brian González-Pérez, Francisco Vázquez-Nava, Josefina Altamira-García, Hannia Rocío González-Quíroz

**Affiliations:** 1 Department of Research, Faculty of Medicine “Dr. Alberto Romo Caballero”, Universidad Autónoma de Tamaulipas, Tampico, Mexico; 2 Family Medicine Service, Family Medicine Unit No. 38, Mexican Social Security Institute (IMSS), Tampico, Mexico

**Keywords:** bioimpedance, body composition, medical interns, physical activity, sleep quality

## Abstract

**Background:**

Becoming a medical intern (MI) represents a major transition in medical education. During this period, trainees are exposed to demanding clinical environments that challenge endurance, adaptability, and self-care. Irregular schedules, sleep deprivation, and reduced opportunities for physical activity (PA) frequently accompany this phase and may lead to measurable changes in anthropometric and body composition (BC) parameters. This study aimed to evaluate one-year changes in BC, PA, and sleep quality (SQ) among MIs and to explore their interrelationship.

**Methods:**

A longitudinal observational study was conducted in 170 MIs (18–25 years) at a Mexican public university hospital, assessed at baseline and after 12 months. Anthropometric and BC parameters were obtained using multifrequency bioimpedance analysis (InBody 270S). PA was assessed with the Global Physical Activity Questionnaire (GPAQ), and SQ with the Pittsburgh Sleep Quality Index (PSQI). Group comparisons were performed using parametric or non-parametric tests as appropriate, with effect sizes reported as Cohen's d and Cramér's V. Statistical significance was set at *p* < 0.05.

**Results:**

Participants had a mean age of 22.9 ± 0.87 years; 62.0% were female. Body mass index (BMI) increased from 25.47 ± 5.06 to 26.22 ± 5.31 kg/m² over the follow-up period. Total body water (TBW), protein mass (PM), and mineral mass (MM) showed relative reductions, while body fat mass (BFM) increased, and skeletal muscle mass (SMM) remained largely stable. The proportion of MIs classified in the low PA category decreased, whereas those engaging in high PA increased. Overall SQ deteriorated, with fewer participants reporting good SQ and a higher proportion classified as poor SQ. Males demonstrated greater increases in BFM, whereas females exhibited relatively more favorable SQ profiles. No significant association was observed between PA level and PSQI-defined SQ.

**Conclusions:**

Despite modest improvements in PA patterns, MIs experienced worsening SQ and unfavorable shifts in BC during the internship year. These findings underscore the vulnerability of medical interns to lifestyle-related health changes and highlight the need for institutional wellness strategies focused on sleep hygiene, balanced nutrition, and sustained opportunities for PA.

## 1 Introduction

The transition from medical student to medical intern (MI) represents one of the most demanding and consequential phases of medical education. Internship is a mandatory, time-limited stage that bridges undergraduate medical training and independent clinical practice, during which trainees become fully integrated into healthcare services and assume supervised clinical responsibilities across hospital settings and, in many contexts, primary care environments (1).

Beyond an individual academic milestone, internship constitutes a systems-level exposure shaped by institutional scheduling and workload structures. As MIs shift from predominantly academic activities to sustained participation in clinical care, they are confronted with irregular schedules, prolonged duty hours, variable workloads, and persistent cognitive and psychological demands. Collectively, these conditions more closely resemble occupational shift work than traditional educational training and define a concentrated period of heightened exposure to work-related health risks within medical education (1, 2). From this perspective, MI health should be conceptualized not solely as an individual attribute, but as a system-sensitive outcome shaped by the structural characteristics of training environments, with direct relevance to institutional priorities such as clinical performance, learning efficiency, and long-term workforce sustainability (3).

Within this framework, the availability of structured transition and support mechanisms may exert a meaningful influence on health-related behaviors (3). In Mexico and other Latin American training settings, limited institutional support across the continuum from pre-clinical education to internship may further exacerbate vulnerability to sedentary behavior, irregular sleep patterns, and suboptimal dietary routines (4, 5). Evidence from university and medical student populations consistently demonstrates a high prevalence of unfavorable lifestyle behaviors during training, including poor sleep quality and low levels of physical activity (2, 4, 5). However, this evidence is largely derived from student-centered, cross-sectional samples and may not adequately capture the magnitude or trajectory of behavioral change during internship, a phase marked by intensified clinical responsibility and greater schedule irregularity (6–10).

Health-related behaviors during internship are not isolated phenomena but instead reflect tightly interwoven physiological and behavioral processes. Diet quality and diversity are central determinants of metabolic status, and inadequate intake patterns during adolescence and young adulthood may promote cardiometabolic risk trajectories that extend into later life (11–13). Sleep is frequently disrupted during medical training, with reduced duration and poorer sleep quality (SQ), often compounded by stress, anxiety, and depressive symptoms (6–9). Importantly, physical activity (PA), SQ, and body composition (BC) are conceptually and biologically linked through shared circadian, neuroendocrine, and behavioral pathways. Under conditions of sustained workload and limited recovery, disruptions in sleep and dietary patterns may attenuate the expected metabolic and body composition benefits of PA, while physical inactivity may further impair SQ, reinforcing adverse health trajectories (6–13).

Despite this conceptual interdependence, the literature on medical trainees remains dominated by cross-sectional designs focused primarily on student populations, limiting the capacity to assess within-individual change and to distinguish transient adaptation from sustained deterioration across the intern year (14, 15). Longitudinal evidence capturing concurrent changes in PA, SQ, and BC during internship remains scarce, particularly in low- and middle-income settings. Internship therefore represents a time-bounded, high-exposure context in which longitudinal follow-up can clarify within-individual trajectories and their interrelationship. In this context, the present study aimed to evaluate one-year changes in BC, PA, and SQ among medical interns, and to explore the interrelationship among these domains across the internship period.

## 3 Methods

### 3.1 Design

We conducted a longitudinal, observational study involving undergraduate MIs aged 18–25 years from the Faculty of Medicine of Tampico “Dr. Alberto Romo Caballero,” enrolled during the 2024-1 academic period. All participants were actively completing their mandatory clinical internship at the time of enrollment. Students were evaluated for anthropometric characteristics, BC, PA levels, and SQ using validated and standardized instruments. Exclusion criteria included any prior diagnosis of medical conditions known to significantly alter BC or sleep physiology, such as endocrine disorders (e.g., hypothyroidism, Cushing's syndrome), chronic inflammatory diseases, or the use of pharmacological agents affecting metabolism, fluid balance, or sleep patterns (e.g., corticosteroids, antipsychotics, hypnotics, or antidepressants). The study protocol was reviewed and approved by the Institutional Review Board of the Faculty of Medicine, Universidad Autónoma de Tamaulipas, approval number UAT-TR-43-22. All participants provided written informed consent prior to enrollment. Measurements were conducted at two time points, baseline and after a 12-month follow-up, to assess intra-individual changes in key physiological and behavioral parameters over time.

### 3.2 Variable measurement

#### 3.2.1 Anthropometric and BC

Anthropometric and BC assessments were performed by trained personnel using the InBody 270S digital scale (InBody Co., Ltd., Seoul, Korea), a validated bioelectrical impedance analyzer that provides a detailed evaluation of body compartments. Measurements were conducted following a standardized protocol and under direct supervision. Participants were evaluated in the morning, barefoot, and in a fasting state, and were instructed to avoid fluid intake, alcohol consumption, and PA for at least 8 h prior to assessment. Height was measured to the nearest 0.1 cm using a portable stadiometer (Seca®, Hamburg, Germany), with participants standing upright and barefoot. The InBody 270S analyzer determines multiple parameters of BC, including body mass (BM) kg, total body water (TBW) kg, protein mass (PM) kg, mineral mass (MM) kg, body fat mass (BFM) kg, skeletal muscle mass (SMM) kg, body mass index (BMI), fitness score, and values for weight, fat, and muscle control (16).

Bioelectrical impedance analysis (BIA) is a noninvasive method based on the conductive properties of body tissues. Water-rich compartments such as muscle conduct electrical current more effectively, while adipose tissue acts as a resistor. This principle allows for the estimation of BC by analyzing resistance to electrical flow, particularly for quantifying FFM and, by subtraction, BFM (17, 18).

#### 3.2.2 Physical activity (PA)

PA was assessed using the Global Physical Activity Questionnaire (GPAQ), developed by the World Health Organization (WHO) to evaluate PA across three domains: occupational, transport-related, and leisure-time activities. The GPAQ consists of 16 items that capture the frequency (days per week) and duration (minutes per day) of moderate- and vigorous-intensity PA, as well as sedentary behavior measured by average daily sitting time. The questionnaire was self-administered, with standardized instructions provided by trained research personnel prior to completion, and responses were reviewed at the time of data collection to minimize missing or inconsistent data. Total PA was expressed in metabolic equivalent minutes per week (MET-min/week), and participants were categorized according to WHO guidelines as having low (<600 MET-min/week), moderate (600–1,499 MET-min/week), or high (≥1,500 MET-min/week) levels of PA (19).

#### 3.2.3 Sleep quality (SQ)

SQ was assessed using the Spanish version of the Pittsburgh Sleep Quality Index (PSQI). The PSQI was self-administered under supervised conditions and evaluates subjective SQ over the preceding month. The instrument comprises 19 items grouped into seven components, each scored from 0 to 3. Component scores were summed to generate a global score ranging from 0 to 21, with higher scores indicating poorer SQ. Global scores were categorized as good (0–4), poor (5–10), or severe sleep disturbance (>10), following established criteria (20, 21).

### 3.3 Statistical analysis

All data were coded and analyzed using IBM SPSS Statistics version 23.0 (IBM Corp., Armonk, NY, USA). Descriptive statistics were used to summarize the characteristics of the participants, with quantitative variables expressed as means and standard deviations (SD) and categorical variables as frequencies and percentages. The Shapiro–Wilk test was applied to assess the normality of continuous variables. For comparisons between groups, the chi-square (*χ*²) test was used for categorical variables, while the Student's *t*-test or Mann–Whitney *U* test was applied for continuous variables, depending on the distribution. Effect sizes were reported using Cohen's d for continuous variables and Cramér's V for categorical comparisons. A *p*-value of <0.05 was considered statistically significant, and all statistical tests were two-tailed. The 95% confidence intervals (CIs) were reported to estimate the precision of the findings

## 4 Results

A total of 170 undergraduate MIs were included in the analysis (mean age 22.9 ± 0.87 years), with a predominance of females (62.0%). Over the one-year follow-up, height remained unchanged, while body weight and BMI showed a modest upward trend (Table 1). BC analysis demonstrated an increase in body fat mass (BFM), accompanied by relative reductions in hydration- and lean tissue–related parameters. SMM remained largely stable throughout the study period (Table 1).

**Table 1:** Descriptive characteristics, body composition, physical activity, and sleep quality of undergraduate medical interns at baseline and one-year follow-up.

Variable	Basal	1 year
Age, year mean + SD (range)	22.9 + 0.87 (22–27)	23.9 + 0.87 (23–28)
Sex, *n* (ratio)		
Female	106 (62.0%)	106 (62.0%)
Male	64 (38.0%)	64 (38.0%)
Height, m mean (range)	1.66 + 0.08 (1.47–1.86)	1.66 + 0.08 (1.47–1.86)
Weight, kg mean (range)	70.64 + 16.55 (40.1–136.1)	72.76 + 17.40 (41.50–133.10)
BMI, kg/m2 mean + SD	25.47 + 5.06 (15.4–47.7)	26.22 + 5.31 (15.20–46.60)
TBW, kg mean + SD	34.85 + 7.85 (19.30–54.10)	35.46 + 8.13 (20.10–55.70)
Protein, kg mean + SD	9.38 + 2.14 (5.09–14.60)	9.53 + 2.26 (5.30–15.30)
Minerals, kg mean + SD	3.34 + 0.68 (2.00–5.13)	3.38 + 0.72 (2.00–5.40)
BFM, kg mean + SD	22.64 + 10.08 (3.27–72.4)	24.48 + 10.67 (5.30–70.90)
SMM, kg mean + SD	26.22 + 6.72 (10.8–42.2)	26.73 + 7.11 (10.90–43.60)
PA, MET-min/week		
Low activity	71 (41.5%)	53 (31.0%)
Moderate activity	51 (29.8%)	60 (35.1%)
High activity	49 (28.7%)	58 (33.9%)
PSQI score		
0–4 points	112 (65.5%)	78 (45.6%)
5–10 points	55 (32.2%)	92 (53.8%)
*>*10 points	4 (2.3%)	1 (0.6%)

BMI, body mass index; TBW, total body water; BFM, body fat mass; SMM, skeletal muscle mass; PA, physical activity; MET, metabolic equivalent of task; PSQI, Pittsburgh sleep quality index; SD, standard deviation.

With respect to physical activity PA, a redistribution across activity categories was observed. The proportion of MIs classified as having low PA decreased at follow-up, while moderate and high activity categories increased. In contrast, SQ worsened at the cohort level over time. The proportion of participants with good SQ declined, whereas poor SQ became more prevalent. Severe sleep disturbances remained infrequent at both assessment points (Table 1).

Sex-based analyses showed no statistically significant differences in BMI between females and males at baseline or at one-year follow-up. However, females exhibited higher absolute body weight at both time points. BC parameters demonstrated sex-related differences across the study period. Females consistently showed higher values in hydration- and lean tissue–related components, whereas males demonstrated a greater increase in BFM over time. SMM differed significantly between sexes at both assessments but remained stable within each sex over the follow-up period (Table 2).

**Table 2:** Sex-based comparison of anthropometric, body composition, physical activity, and sleep parameters at baseline and one-year follow-up.

Variable	Baseline	*P value*	1 year	*P value*
Weight, kg mean (range)		0		0
Female	78.8 + 15.29		80.7 + 16.73	
Male	65.6 + 15.31		67.8 + 16.02	
BMI, kg/m^2^ mean + SD		0.24		0.49
Female	26.0 + 4.69		26.5 + 5.02	
Male	25.1 + 5.26		26.0 + 5.49	
TBW, kg mean + SD		0.03		0
Female	42.4 + 5.82		43.00 + 6.45	
Male	30.2 + 4.72		30.84 + 5.00	
Protein, kg mean + SD		0.04		0.01
Female	11.4 + 1.59		11.6 + 1.83	
Male	8.1 + 1.29		8.23 + 1.34	
Minerals, kg mean + SD		0.03		0
Female	3.9 + 0.57		3.9 + 0.57	
Male	2.9 + 0.45		3.0 + 0.49	
BFM, kg mean + SD		0.14		0.01
Female	21.2 + 9.97		21.9 + 10.29	
Male	23.5 + 0.87		26.0 + 10.65	
SMM, kg mean + SD		0.03		0.03
Female	32.6 + 5.01		33.3 + 5.36	
Male	22.3 + 4.13		22.6 + 4.54	
PA, MET-min/week		0.37		0.62
Female				
Low activity	16 (24.6%)		23 (35.4%)	
Moderate activity	22 (33.8%)		21 (32.3%)	
High activity	27 (41.5%)		21 (32.3%)	
Male				
Low activity	35 (33.0%)		30 (28.3%)	
Moderate activity	27 (25.5%)		39 (36.8%)	
High activity	44 (41.5%)		37 (34.9%)	
PSQI score				
Female		0		0.49
0–4 points	15 (23.1%)		27 (41.5%)	
5–10 points	46 (70.8%)		38 (58.5%)	
*>*10 points	4 (6.2%)		0 (0.0%)	
Male				
0–4 points	40 (37.7%)		51 (48.1%)	
5–10 points	66 (62.3%)		54 (50.9%)	
*>*10 points	0 (0.0%)		1 (0.9%)	

BMI, body mass index; TBW, total body water; BFM, body fat mass; SMM, skeletal muscle mass; PA, physical activity; MET, metabolic equivalent of task; PSQI, Pittsburgh sleep quality index; SD, standard deviation; P, *p* value obtained using student's *t* test for quantitative variables and *X*² test for categorical variables.

PA distribution did not differ significantly by sex at baseline or follow-up. Nonetheless, opposing trends were observed: females showed a shift toward lower activity levels, while males demonstrated a redistribution toward moderate activity. In sex-stratified analyses, SQ displayed distinct patterns. Females exhibited a reduction in severe sleep disturbances over time, which were absent at follow-up, whereas overall SQ categories remained largely unchanged among males (Table 2). These findings indicate differential adaptations in BC, PA behavior, and sleep patterns between sexes during the internship year (Table 2).

To evaluate the relationship between PA level and SQ, participants were stratified according to PA category and PSQI-defined SQ at baseline and follow-up (Table 3). At baseline, no statistically significant association was observed between PA level and SQ, and PSQI categories were similarly distributed across low, moderate, and high PA groups. A comparable pattern was observed at one-year follow-up, with no significant differences in SQ distribution across PA levels. Although descriptive changes in SQ occurred over time, PA level did not differentiate SQ status at either assessment point (Table 3).

**Table 3:** Distribution of sleep quality (PSQI scores) by physical activity level at baseline and one-year follow-up.

	Physical activity level			
PSQI category	Low	Moderate	High	*P value*
Baseline PSQI score				
0–4 points	19 (37.3%)	18 (20.4%)	26 (36.6%)	0.11
5–10 points	30 (58.8%)	37 (75.5%)	45 (63.4%)	0.19
*>*10 points	2 (3.9%)	2 (4.1%)	0 (0.0%)	0.23
1 year PSQI score				
0–4 points	24 (45.3%)	27 (45.0%)	27 (46.6%)	0.98
5–10 points	28 (52.8%)	33 (55.0%)	31 (53.4%)	0.97
*>*10 points	0 (0.0%)	1 (1.9%)	0 (0.0%)	0.32

PSQI, Pittsburgh sleep quality index; P, *p* value obtained using *X*² test.

## 5 Discussion

The interconnection between SQ, levels of PA, and BC among MIs warrants interpretation within the specific framework of internship training, a phase characterized by elevated workload, persistent stress, and substantial cognitive demands. Although obesity-related studies in medical students are limited, available evidence indicates that excess adiposity is present even in this population, with body fat percentages varying across countries according to genetic background, dietary patterns, habitual PA, and other lifestyle factors (22).

In this context, the present findings underscore the limitations of relying on BMI alone, as BMI does not adequately reflect the metabolic, cardiovascular, and other health risks associated with increased body fat (23). The observed increases in body weight and BFM in this young cohort highlight the importance of direct BC assessment, as it captures the cumulative impact of lifestyle behaviors and training-related exposures during internship. Notably, these unfavorable BC changes occurred despite improvements in PA, suggesting that, within the internship setting, increased activity alone may be insufficient to counterbalance the multifactorial determinants of BC. Dietary inadequacy, sleep deprivation, sustained psychological stress, and prolonged sedentary behavior likely attenuate the expected metabolic benefits of PA, particularly under conditions of high training demand (24, 25).

Prolonged sedentary behavior during internship may exert independent metabolic effects, including impaired glycemic regulation, reinforcing the relevance of limiting uninterrupted sitting time even among young and apparently healthy individuals (26). At the same time, extended academic and clinical workloads restrict opportunities for regular, structured physical activity, thereby increasing vulnerability to excess adiposity among medical trainees (27). These constraints are frequently compounded by inadequate or imbalanced dietary patterns, which are prevalent among university populations and may further exacerbate unfavorable BC trajectories during training (28, 29).

Regarding sleep outcomes, PA level alone did not significantly differentiate SQ during internship training, as no statistically significant association was observed between PA levels and PSQI-defined SQ. Although descriptive trends suggested more favorable sleep profiles among interns engaging in higher levels of PA, these patterns did not translate into measurable differences. This contrasts with findings from broader university populations, in which positive associations between PA and SQ have been more consistently reported (30, 31), as well as in adolescents and adult populations across different age groups (32–35). Together, these observations suggest that, among MIs, the potential sleep-related benefits of PA may be attenuated by dominant training-related factors, including high workload intensity, psychological stress, irregular schedules, and restricted sleep opportunity.

The absence of a significant association between PA and SQ in the present study highlights the multifactorial regulation of sleep during internship, where PA alone may be insufficient to offset the effects of prolonged sedentary behavior, academic demands, night work exposure, and circadian disruption (29, 36). Importantly, the heterogeneous sleep adaptations observed-characterized by overall deterioration in SQ at the cohort level alongside sex-specific improvements in severe sleep disturbances-suggest that sleep regulation during the internship is not uniform and may reflect differential vulnerability or adaptive responses within this population (37, 38). In this context, while the promotion of PA among MIs remains essential given its well-established benefits for physical health, mental well-being, burnout mitigation, and professional role modeling, sleep-related outcomes during internship require cautious and nuanced interpretation.

SQ is critical to the well-being and academic performance of students in demanding health professions such as nursing and medicine, where intensive schedules, clinical responsibilities, and sustained academic stress are common. The relatively young age of the present cohort is relevant, as young adulthood is a developmental stage characterized by lifestyle transitions, heightened academic and social pressures, and increased exposure to technology-related sleep disruption, all of which may predispose individuals to irregular sleep schedules, delayed sleep onset, and insufficient sleep duration (39).

Within this broader context, the persistently poor SQ observed in the present cohort, together with heterogeneous sex-specific adaptations, aligns with prior reports and underscores the vulnerability of MIs during this training phase (29). Although underlying mechanisms were not directly assessed, existing evidence indicates that sleep disruption and circadian misalignment during early adulthood are associated with metabolic dysregulation, including alterations in appetite regulation, increased caloric intake, and unfavorable BC trajectories (30). These mechanisms may help contextualize the concurrent patterns of poor SQ and increased BFM observed in this study. Moreover, circadian rhythm disturbances related to irregular work schedules and disrupted sleep–wake cycles have been linked to impaired coordination of physiological processes, contributing to both physical and mental health consequences (34). Epidemiological evidence further supports an association between inadequate sleep and increased obesity risk across populations (35). Collectively, these findings reinforce sleep disruption during internship as a meaningful contextual factor with potential implications for metabolic health, cognitive performance, and overall well-being in medical trainees (40).

This study has several limitations that should be considered when interpreting the findings. First, key lifestyle variables, including PA, SQ, and dietary behaviors, were assessed using self-reported questionnaires, which may be subject to recall and social desirability bias. Objective assessments of SQ (e.g., actigraphy) and diet quality were not available, potentially limiting measurement precision. Second, although BC and BFM were assessed using bioelectrical impedance analysis, hydration status and recent PA may have influenced certain parameters despite standardized measurement procedures. Third, the observational design precludes causal inference regarding the relationships between PA, SQ, and BC. Additionally, potential confounders such as type of clinical rotation, shift work characteristics, dietary patterns, workload intensity, and psychosocial stress were not fully controlled. Finally, the study was conducted in a single cohort of MIs, which may limit the generalizability of the findings to other trainee populations or training settings. Nevertheless, these limitations also emphasize the relevance of contextual and structural factors inherent to internship training, suggesting that observed health-related patterns reflect not only individual behaviors but also modifiable characteristics of the training environment. Together, these considerations underscore the need for future longitudinal studies incorporating objective assessments and more detailed characterization of training-related contextual variables.

## 6 Conclusions

During the internship year, medical trainees exhibited unfavorable trends in body composition, characterized by increased body fat and BMI, along with a decline in hydration and cellular mass parameters, despite slight improvements in PA levels. At the cohort level, SQ remained suboptimal over time, although heterogeneous patterns were observed, including a modest reduction in severe sleep disturbances in specific subgroups. These findings suggest that increases in PA may coexist with concurrent changes in BC and SQ in the context of irregular schedules, prolonged shifts, and chronic stress typical of internship training. Sex-specific differences were evident, with males showing greater fat accumulation and females reporting relatively more favorable sleep-related outcomes, without implying overall SQ improvement at the population level. Collectively, these results underscore the vulnerability of medical interns to lifestyle-related alterations during this demanding training phase and highlight the potential value of institution-level wellness strategies targeting sleep hygiene, nutritional habits, and structured opportunities for PA. Incorporating periodic monitoring of BC and SQ into training programs may facilitate early identification of emerging health risk patterns and support preventive approaches aimed at preserving trainee well-being.

## Data Availability

The original contributions presented in the study are included in the article/Supplementary Material, further inquiries can be directed to the corresponding author.
