# Supplementary material for: Medical interns and health challenges: insights into physical inactivity, sleep disruption, and body metrics
Source: Front Health Serv. 2026 Mar 18;6:1735424. doi: 10.3389/frhs.2026.1735424 (PMC13038937; doi:10.3389/frhs.2026.1735424)
Supplement: Supplementary file 1 [file Table1.pdf]

Table 1: Descriptive Characteristics, Body Composition, Physical Activity, and Sleep Quality of Undergraduate Medical Interns at Baseline and One-Year Follow-Up

	Basal	1 year
Age, year mean + SD (range)	22.9 + 0.87 (22-27)	23.9 + 0.87 (23-28)
Sex, n (ratio)		
Female	106 (62.0 %)	106 (62.0 %)
Male	64 (38.0 %)	64 (38.0 %)
Height, m mean (range)	1.66 + 0.08 (1.47-1.86)	1.66 + 0.08 (1.47-1.86)
Weight, kg mean (range)	70.64 + 16.55 (40.1-136.1)	72.76 + 17.40 (41.50-133.10)
BMI, kg/m2 mean + SD	25.47 + 5.06 (15.4 - 47.7)	26.22 + 5.31 (15.20-46.60)
TBW, kg mean + SD	34.85 + 7.85 (19.30-54.10)	35.46 + 8.13 (20.10-55.70)
Protein, kg mean + SD	9.38 + 2.14 (5.09-14.60)	9.53 + 2.26 (5.30-15.30)
Minerals, kg mean + SD	3.34 + 0.68 (2.00-5.13)	3.38 + 0.72 (2.00-5.40)
BFM, kg mean + SD	22.64 + 10.08 (3.27-72.4)	24.48 + 10.67 (5.30-70.90)
SMM, kg mean + SD	26.22 + 6.72 (10.8-42.2)	26.73 + 7.11 (10.90-43.60)
PA, MET-min/week		
Low activity	71 (41.5 %)	53 (31.0 %)
Moderate activity	51 (29.8 %)	60 (35.1 %)
High activity	49 (28.7 %)	58 (33.9 %)
PSQI score		
0 - 4 points	112 (65.5 %)	78 (45.6 %)
5 - 10 points	55 (32.2 %)	92 (53.8 %)
>10 points	4 (2.3 %)	1 (0.6 %)

BMI: Body mass index, TBW: total body water, BFM: Body Fat Mass, SMM: Skeletal muscle mass, PA: Physical activity, MET: Metabolic equivalent of task, PSQI: Pittsburgh Sleep Quality Index, SD: Standar deviation.
