# Supplementary material for: Medical interns and health challenges: insights into physical inactivity, sleep disruption, and body metrics
Source: Front Health Serv. 2026 Mar 18;6:1735424. doi: 10.3389/frhs.2026.1735424 (PMC13038937; doi:10.3389/frhs.2026.1735424)
Supplement: Supplementary file 2 [file Table2.pdf]

Table 2. Sex-Based Comparison of Anthropometric, Body Composition, Physical Activity, and Sleep Parameters at Baseline and One-Year Follow-Up

	Baseline	P	1 year	P
Weight, kg mean (range)		0		0
Female	78.8 + 15.29		80.7 + 16.73	
Male	65.6 + 15.31		67.8 + 16.02	
BMI, kg/m2 mean + SD		0.24		0.49
Female	26.0 + 4.69		26.5 + 5.02	
Male	25.1 + 5.26		26.0 + 5.49	
TBW, kg mean + SD		0.03		0
Female	42.4 + 5.82		43.00 + 6.45	
Male	30.2 + 4.72		30.84 + 5.00	
Protein, kg mean + SD		0.04		0.01
Female	11.4 + 1.59		11.6 + 1.83	
Male	8.1 + 1.29		8.23 + 1.34	
Minerals, kg mean + SD		0.03		0
Female	3.9 + 0.57		3.9 + 0.57	
Male	2.9 + 0.45		3.0 + 0.49	
BFM, kg mean + SD		0.14		0.01
Female	21.2 + 9.97		21.9 + 10.29	
Male	23.5 + 0.87		26.0 + 10.65	
SMM, kg mean + SD		0.03		0.03
Female	32.6 + 5.01		33.3 + 5.36	
Male	22.3 + 4.13		22.6 + 4.54	
PA, MET-min/week		0.37		0.62
Female				
Low activity	16 (24.6 %)		23 (35.4 %)	
Moderate activity	22 (33.8 %)		21 (32.3 %)	
High activity	27 (41.5 %)		21 (32.3 %)	
Male				
Low activity	35 (33.0 %)		30 (28.3 %)	
Moderate activity	27 (25.5 %)		39 (36.8 %)	
High activity	44 (41.5 %)		37 (34.9 %)	
PSQI score				
Female		0		0.49
0 - 4 points	15 (23.1 %)		27 (41.5 %)	
5 - 10 points	46 (70.8 %)		38 (58.5 %)	
>10 points	4 (6.2 %)		0 (0.0 %)	
Male				
0 - 4 points	40 (37.7 %)		51 (48.1 %)	
5 - 10 points	66 (62.3 %)		54 (50.9%)	
>10 points	0 (0.0 %)		1 (0.9 %)	

BMI: body mass index; TBW: total body water; BFM: body fat mass; SMM: skeletal muscle mass; PA: physical activity; MET: metabolic equivalent of task; PSQI: Pittsburgh Sleep Quality Index; SD: standard deviation; P: p value obtained using Student's t test for quantitative variables and X^2 test for categorical variables.
