# Supplementary material for: Medical interns and health challenges: insights into physical inactivity, sleep disruption, and body metrics
Source: Front Health Serv. 2026 Mar 18;6:1735424. doi: 10.3389/frhs.2026.1735424 (PMC13038937; doi:10.3389/frhs.2026.1735424)
Supplement: Supplementary file 3 [file Table3.pdf]

Table 3. Distribution of Sleep Quality (PSQI Scores) by Physical Activity Level at Baseline and One-Year Follow-Up

	Physical activity level			
	Low	Moderate	High	P
Baseline PSQI score				
0 - 4 points	19 (37.3 %)	18 (20.4 %)	26 (36.6 %)	0.11
5 - 10 points	30 (58.8 %)	37 (75.5 %)	45 (63.4 %)	0.19
>10 points	2 (3.9 %)	2 (4.1 %)	0 (0.0 %)	0.23
1 year PSQI score				
0 - 4 points	24 (45.3 %)	27 (45.0 %)	27 (46.6 %)	0.98
5 - 10 points	28 (52.8 %)	33 (55.0 %)	31 (53.4 %)	0.97
>10 points	0 (0.0 %)	1 (1.9 %)	0 (0.0 %)	0.32

PSQI: Pittsburgh Sleep Quality Index; P: p value obtained using X^2 test.
